# Recent Progress in Metabolic Syndrome Research and Therapeutics

**DOI:** 10.3390/ijms22136862

**Published:** 2021-06-25

**Authors:** Ting-Wei Kao, Chin-Chou Huang

**Affiliations:** 1 Department of Internal Medicine, National Taiwan University Hospital, Taipei 100, Taiwan; twkao315@gmail.com; 2 Division of Cardiology, Department of Medicine, Taipei Veterans General Hospital, Taipei 112, Taiwan; 3 School of Medicine, National Yang Ming Chiao Tung University, Taipei 112, Taiwan; 4 Cardiovascular Research Center, National Yang Ming Chiao Tung University, Taipei 112, Taiwan; 5 Institute of Pharmacology, National Yang Ming Chiao Tung University, Taipei 112, Taiwan

**Keywords:** metabolic syndrome, metabolomics, microbiota, precision medicine, proteomics

## Abstract

Metabolic syndrome (MetS) is a well-defined yet difficult-to-manage disease entity. Both the precipitous rise in its incidence due to contemporary lifestyles and the growing heterogeneity among affected populations present unprecedented challenges. Moreover, the predisposed risk for developing severe acute respiratory syndrome coronavirus 2 (SARS-CoV-2) infection in populations with MetS, and the viral impacts on host metabolic parameters, underscores the need to investigate this mechanism thoroughly. Recent investigations of metabolomics and proteomics have revealed not only differentially expressed substances in MetS, but also the consequences of diet consumption and physical activity on energy metabolism. These variations in metabolites, as well as protein products, also influence a wide spectrum of host characteristics, from cellular behavior to phenotype. Research on the dysregulation of gut microbiota and the resultant inflammatory status has also contributed to our understanding of the underlying pathogenic mechanisms. As for state-of-the-art therapies, advancing depictions of the bio-molecular landscape of MetS have emerged and now play a key role in individualized precision medicine. Fecal microbiota transplantation, aiming to restore the host’s homeostasis, and targeting of the bile acid signaling pathway are two approaches to combatting MetS. Comprehensive molecular inquiries about MetS by omics measures are mandatory to facilitate the development of novel therapeutic modalities.

## 1. Introduction

Metabolic syndrome (MetS) is classically recognized as a cluster of at least three of the five following conditions: central obesity, hypertension, hyperglycemia, hypertriglyceridemia, and low serum high-density lipoprotein [1,2,3]. The etiologies are proposed to be multi-factorial, including diet pattern, genetic predisposition, ethnicity, etc. [4]. A genetic risk score, based on a Korean cohort database, was previously proposed to demonstrate the interplay between physical activity and MetS risk in different genetic backgrounds by assessing four established single nucleotide polymorphisms [5]. The MEDLIFE index is an assessment based on Mediterranean lifestyle, including food choice, exercise, and social interaction. Adherence to Mediterranean lifestyle has been shown to decrease the incidence of MetS [6]. Although ascertaining the exact prevalence of MetS remains a challenge, epidemiological studies have suggested that over one billion individuals worldwide suffer from MetS, and the value is in constant escalation, given lifestyle modifications secondary to urbanization in modern society [7]. Children and adolescents in high income regions may be especially prone to MetS [8], marking it a significant clinical, as well as social, issue of concern.

Holistic care of MetS patients is essential as these patients are predisposed to a variety of cardiovascular, cerebral, and hepato-renal complications, as well as increased all-cause mortality [9,10,11,12,13,14,15]. There is a strong association between MetS and type 2 diabetes mellitus (DM) [16]. In addition, as MetS elevates apo B-containing lipoprotein to drive atherogenesis and lesion formation, atherosclerosis is a frequent comorbidity of MetS [17]. The clinical characteristics of MetS subsequently induce plaque rupture and bring about thromboembolic event. Multi-site arterial calcification observed in MetS patients has been shown to predict cardiovascular events and coronary disease. Recent investigations have shed light into the underlying pathogenesis of this process [18]. In addition to established cardiovascular mortality, the presence of MetS is correlated with an increased risk of sudden cardiac death, as shown by Hess et al., who analyzed 13,168 American patients from the Atherosclerosis Risk in Communities Study database with 23.6 years of follow up. Furthermore, susceptibility to sudden death is increased when more MetS criteria are met, regardless of gender or ethnicity [19]. Both elevation in body mass index (BMI) and body weight gain are independent risk factors for the development of MetS [20]. MetS also disposes individuals to systematic adverse events. Glomerular hyperfiltration, eventual chronic kidney disease, and excessive mortality rate, were found to be associated with non-alcoholic fatty liver disease in MetS hosts [21,22]. Timely diagnosis, prompt management, and early prevention are the clinical goals to approaching cases of MetS.

In the recent severe acute respiratory syndrome coronavirus 2 (SARS-CoV-2) pandemic, MetS was suggested to be a risk factor for viral infection and increased rate of severe complications. Such observations prompted further investigation of the relationship between MetS and coronavirus. However, conventional strategies for assessing MetS have seldom addressed the bio-molecular variation emergent in the disease’s evolution, which has limited our understanding of the endogenous heterogeneity of MetS. In this review, we pursued a bottom-up approach to summarize recent progress in MetS research. We reviewed novel findings regarding MetS and SARS-CoV-2, metabolomics and proteomics studies, and gut microbiota, as well as the discussion of novel therapeutic modalities targeting the molecular variation in MetS, so as to enhance the practice of individualized precision medicine against metabolic disorders.

## 2. MetS and SARS-CoV-2

Individuals with MetS often have several pathological comorbidities, and the global pandemic of SARS-CoV-2 ignited the concern for the predisposed risk of infection in this population. In addition to traditional cardiovascular risk factors, including advanced age, male, obesity, elevated blood pressure, and DM, the entity of MetS was investigated in the setting of coronavirus infection. A Chinese observational study reported those with underlying cardio-metabolic illness harbored a greater risk of aggravation to critical condition, which could worsen their eventual prognosis [23]. In one Mexican epidemiological investigation analyzing 528,651 subjects, mortality rates doubled with the addition of each MetS criteria [24]. As for Europeans, the study involving 9005 subjects from the UK Biobank coherently suggested elevated BMI and DM were associated with increased susceptibility of SARS-CoV-2 infection [25]. Regardless of ethnic background, MetS became the central issue of concern during the pandemic. Investigations toward the correlation between obesity and SARS-CoV-2 infection, as well as its mortality, were thereby carried out. Central players relevant to this association were angiotensin converting enzyme 2 receptor (ACE2R) and lipid, in conjunction with intertwined chemokines. Highly expressed by adipocytes, ACE2R served as the essential gate for virus to enter cell [26], causing direct damage. Another aspect is the indirect effect, which refers to the immunological activation caused by the viral infection and its deleterious consequences on the host. Lipid, however, was proposed to be a reservoir of proinflammatory mediators, e.g., tumor necrosis factor α, monocyte chemoattractant protein 1, and interleukin-6. An observational study reported enhanced susceptibility of SARS-CoV-2 infection in patients with metabolic associated fatty liver disease. Further investigations suggested obesity was intertwined with compromised immunity in the context of a hyperinflammatory state [27]. The metabolic perturbation could even initiate cytokine storm, leading to impaired fatty acid oxidation, diminished gluconeogenesis, and subsequent acute kidney injury [28]. SARS-CoV-2 was also shown to alter the metabolic status of the host through peroxisome proliferator-activated receptor. These delineated the close relationship between metabolism and inflammation.

The endocrine-immune axis not only explains the culprit pathophysiology, but also illuminates potential treatment for infected patients with MetS. Trials with traditional oral hypoglycemic agents are ongoing to validate the efficacy in this cohort. Matrix metalloproteinases were pinpointed to be responsible for the orchestration of tissue inflammation and metabolism of end organs. The hypothesis was therefore raised to enhance metabolic tolerance under SARS-CoV-2 infection by manipulating this target [29]. Finally, the concept of immunometabolic reprogramming was suggested as a potential novel therapeutic modality. Xiao et al. first extensively characterized metabolomics and the expression of interleukin-6, macrophage-colony stimulating factor, interleukin-1α, and interleukin-1β from both healthy volunteers and patients with confirmed SARS-CoV-2 infection [30]. Alteration of the host metabolic profile was shown to impact the abundance of cytokine secretion from SARS-CoV-2 cells by affecting suspected mediators such as arginine, tryptophan, and purine, which were raised to be the players involved. This finding validated the concept of ameliorating systematic inflammation and the potentially ensued cytokine storm by targeting the metabolism. However, the exact mechanism explaining how SARS-CoV-2 interfered with the host metabolic panel remains unknown. Determination of which metabolic parameters to establish risk stratification score or prognostic scores requires further study. As for clinical translation, achieving effective anti-inflammation by metabolic maneuvers may be a feasible treatment strategy both against SARS-CoV-2 pandemic as well as other autoimmune disorders.

## 3. Metabolomics

Metabolomics is a cutting-edge approach to understanding the signal transduction of MetS. Metabolomics refers to the systematic analysis of metabolites in a biological specimen, including sugars, amino acids, organic acids, nucleotides, and lipids [31,32]. Unlike methods designed to determine the role of single players in the entire transduction pathway, metabolomics explores the alteration of molecular landscapes under certain stimuli or pathologic conditions. Both metabolites expressed by the host itself, and derived from endogenous microbiota participated in the signaling pathways and biochemical alterations that govern energy homeostasis [33]. Detailed metabolomic elucidation drives the progression of biomarker identification, drug discovery [34,35], toxicology [36], and nutrition studies [37] (Table 1). Various analytic modalities were developed to elucidate the complicated and wildly diverse molecular expressions, especially in cases with chronic MetS harboring multifaceted etiologies. 

Recognizing both specific and novel metabolites is crucial to assessing MetS. This kind of experimental platform model was first established by Nordstrom et al. [47], and the analytic strategy for global metabolite profiling of human metabolome was founded [48]. Subsequently, metabolomics and lipidomics delineation by liquid/gas chromatography mass spectrometry (MS) was conducted on 115 middle-aged Dutch individuals (50 with MetS; 65 controls) in the Leiden Longevity Study. Nine metabolites, predominantly acylcarnitines, amino acids, and keto acids, as well as ten types of lipids were disclosed and found to be negatively associated with the MetS score, while 26 metabolites and most types of triacyl-glycerides were positively correlated. In a univariate analysis, valeryl carnitine, pyruvic acid, lactic acid, alanine, and diglyceride were also found to be relevant [38]. Mainly, these molecules were intertwined with the metabolism of glucose, amino acid, and lipid [49]. A distinct metabolite profile was further linked to cardiovascular risk, including androgen, fatty acid, phosphatidylethanolamine, and phophatidylcholine [39]. Polyphenol was proposed in recent decades to be both effective in antioxidation and essential in the maintenance of gut microbiota homeostasis [50]. From a meta-analysis pooling, 16 manuscripts, a total of 361 metabolites in 22 families, were identified to be independently intertwined with at least one component of MetS (27 of which fully associated with all MetS criteria) [51]. Albeit abundant, the excessively complicated repertoire of the metabolite landscape made it challenging to draw conclusions and propel the establishment of practice guidelines. Improvement in the modality for investigations on metabolites, e.g., untargeted methodology and multiplatform analysis, was proposed for novel biomarker identifications.

Additionally, metabolomic characteristics have been proposed to possess prognostic significance. Gall et al. first utilized mass spectrometry, and identified alpha-hydroxybutyrate as an early biomarker of insulin resistance and glucose intolerance [40]. Using untargeted metabolomics, clinical, sociodemographic, and diet parameters, the occurrence of MetS was successfully predicted in the French occupational GAZEL cohort [52], and MS-based metabolomic profiling effectively detected and monitored MetS [41]. In a population-based study, analysis of serum metabolites harvested from 2380 subjects revealed the metabolism of hepatic phospholipid was correlated with cardiopulmonary fitness [42]. Moreover, Libert et al. analyzed the biochemical derangements of plasma metabolomics in 90 obese individuals, and suggested the escalation of branched-chain amino acids (BCAAs), aromatic amino acids, lysine, and alpha-aminoadipate were correlated with worsened metabolic health [43]. Similarly, 13 metabolites preferentially expressed in adult-onset Still’s disease were shown to be MetS-related [44]. Together, analysis of metabolomics not only brought to light early alarms, but also yielded insights into MetS aggravation.

How food intake modifies plasma metabolome is another attractive research topic. Capel et al. interrogated the metabolome of 298 participants (61 with MetS) from the French MONA LISA survey. Intriguingly, some MetS-specific metabolomic variations were mitigated by the consumption of low-fat dairy products [45], and a recent meta-analysis echoed this result, suggesting low fat milk and yogurt ameliorated the risk of MetS [53]. Saponin, a major component of several traditional Chinese medicines, was shown to exert a protective role against MetS [54]. The molecule was also revealed to counteract oxidative stress and revitalize the NO signaling pathway in animal models, thereby enhancing left ventricular ejection fraction in MetS hosts [55]. Other in vitro investigations validated the effect of lipase inhibition, which antagonizes lipogenesis, and anti-obesity effects of saponin. 

Exercise also affects the onset of MetS on the molecular level. Siopi et al. investigated the response of serum metabolic fingerprints to different exercise modes. A total of 23 sedentary males (9 with MetS; 14 healthy participants) were enrolled and underwent four exercise modes. Resistance exercise exerted the strongest beneficial effect, followed by high-intensity interval exercise. The efficacy of continuous moderate-intensity exercise was minimal. In conjunction, several prominent biomarkers, e.g., BCAAs, alanine, acetylcarnitine, choline, and betaine, underpin the development of MetS [46]. These studies provide mechanisms by which reshaping lifestyle has the potential to antagonize MetS.

## 4. Proteomics

The proteome is downstream to genomic and transcriptomic processes, and proteomics is known as the methodical scrutiny of complete constituents of proteins expressed in a biological system under divergent physiological or pathological stimuli. Although the human genome contains approximately 20,300 genes, differential transcription, splicing, amino acid polymorphisms, and post-translational modification (e.g., methylation, acetylation, phosphorylation, and glycosylation) can result in more than 100 different functional products. Similar to metabolomics, proteomic studies uncover the orchestration of the differential abundance of protein products, which thereafter influences the homeostasis of host energy utilization [56]. In conjunction with genomic research, these investigations attempt to elucidate the molecular network and identify possible therapeutic targets. Here, we review the recent advances in proteomic research on MetS (Table 2).

Proteomics is ideal for exploring MetS as it offers a comprehensive approach. An animal study presented the contrasting expression pattern of hepatic proteins between a fructose-induced MetS group versus a healthy control, revealing alterations in glucose and fatty acid metabolic pathways [57]. Similarly, Benade et al. performed a proteomic analysis of liver samples on mice that had consumed sugar-sweetened beverages. Albeit in the presence of adaptive responses, perturbations in mitochondrial physiology predisposed subjects to the development of cardiometabolic comorbidities [58]. Proteomic and transcriptomic profiling were used to delineate the physiology of elder MetS patient. A recent study using muscle biopsy demonstrated dominant proteolysis, diminished oxidative phosphorylation, and impaired clearance of reactive oxygen species in this population [59]. Dicarbonyl stress is also a proposed mechanism for the development of renal microvascular complications [60]. Furthermore, obesity not only phenotypically changed the appearance of adipocytes, but also altered the proteomic signature in cardiac visceral adipose tissue (e.g., enriching the abundance of proteins involved in triglyceride metabolism), leading to myocardial consequences [61]. Distinguishing proteomic alterations is central to understanding the pathogenesis of MetS.

**Table 2:** Study of proteomics. MetS, metabolic syndrome.

Author, Year	Species	Cohort	Findings	Ref
Hsieh, 2016	Animal	Male Sprague Dawley rats	High-fructose diet escalates oxidative stress, disturbing glucose and fatty acid metabolism.	[57]
Benade, 2020		Male Wistar rats	Altered mitochondrial physiology predisposes cardiometabolic complications.	[58]
Markova, 2019		Male hereditary hypertriglyceridaemic rats	Dicarbonyl stress aggravated by methylglyoxal, causing renal dysfunction in MetS.	[60]
Conceição, 2020		ZSF1 rats	Obesity changes the proteomic expression, leading to myocardial consequences.	[61]
Pla-Pagà, 2020		Sprague Dawley rats	Hesperidin supplementation alters proteomic landscape and the risk of corresponding complications.	[62]
Gueugneau, 2021	Human	30 healthy subjects, 9 patients with MetS	MetS promoted proteolysis and antagonizing oxidative phosphorylation.	[59]
Alfadda, 2017		18 Saudi obese patients	Metabolically unhealthy patients present differential levels of 18 proteins.	[63]
Sharma, 2019		80 pre-menopausal females	History of miscarriage is correlated with MetS via transthyretin	[64]

To investigate the influence of protein signatures on cellular behavior, proteomic studies of visceral adipose tissue from metabolically unhealthy obese patients have illustrated that pathways related to cell migration, development of the hematological system, and immune cell trafficking can be drastically impacted [63]. For other comorbidities of MetS, transthyretin was identified as a molecular linkage between miscarriage and MetS [64]. The static status was assessed, the dynamics of proteomic evolvement carries clinical implications as well. For example, supplementation of hesperidin strongly modified protein expression in rodent heart and renal tissues, thereby re-conditioning the risk of downstream complications [62]. Further, exogenous stimuli were documented to be memorized by proteomic imprinting and thereby exerted a legacy effect. Combining transcriptomics and proteomics, these newfound measures offer insights into detailed cellular and molecular mechanisms of MetS.

## 5. Microbiota and Inflammation

The status of inflammation has been well-recognized in MetS, as well as consequential complications established to be the consequence. For example, atherosclerosis was demonstrated to be pathogenically associated with excessive oxidation and inflammation, possibly due to dysfunctional autophagy, and thus compromised intracellular homeostasis in MetS [65]. In recent years, a view that has been gaining attention is that gut disarray is a potential cause of MetS. Although there is argument that the metabolites related to insulin resistance and those associated with cardiovascular prognosis bore little resemblance [39], the effect exerted by altered intestinal microbiomes in response to exogenous stimuli still impacts the host [66]. Emerging evidence also suggests a role in the pathogenesis of MetS. Interestingly, the landscape of gut microbiota was transiently reshaped with diet and steroid-induced imprint of inflammation, hepatic steatosis, and insulin sensitivity in the porcine model. The alteration was recognized to recapitulate the human MetS signature, particularly bile and fatty acid [67]. The peri-implant dental sample harvested from subjects with MetS exhibited remarkably higher level of Aggregatibacter Actinomycetemcomitans, Prevotella intermedia, and Staphylococcus aureus [68], highlighting the systematic significance of metabolic disorder. In this section, we revisit investigations regarding microbiota and inflammation (Table 3). 

Pioneering evidence based on animal models featuring germ-free mice and obese (OB/OB) rodents have demonstrated specific microbial compositions in the setting of MetS, DM, atherosclerosis, non-alcoholic liver disease, hypertension, etc. [77,78]. Impaired intestinal barriers permit the translocation of bacteria and their components from the gastrointestinal lumen into the bloodstream [82,83]. Such metabolic endotoxemia or bacteremia [69] triggers an inflammatory state, along with the activation of c-jun amino-terminal kinase (JNK) [70], peroxisome proliferator-activated receptor gamma (PPARG), and the corresponding nuclear factor kappa-light-chain-enhancer of the activated B cells (NF-kB) signaling pathway [84]. Meanwhile, through the infiltration of gut microbiota, obesity leads to the downregulation of JNK and PPARG, causing insulin resistance. The migration of bacteria into the end organ also results in morbidity secondary to MetS. It remains a challenge to reproduce these findings in humans [85]. However, through the advancement in sample harvesting and viability assessment for bacterial components of interest, MetS was further validated to be highly associated with inflammation in vivo [86]. Future clinical studies, however, are still warranted to further clarify the relationship between inflammation and MetS.

Metabolic disruption is also coordinated via small molecules generated by gut flora. Short-chain fatty acids (SCFA) are produced from hydrolysis and subsequent fermentation of consumed polysaccharides by intestinal microbiota. SCFA affects satiety through peptide YY and glucagon-like peptide 1 (GLP-1) [71,72]. In addition, SCFA is involved in fatty acid oxidation, lipogenesis, and energy utilization [73]. Interestingly, downstream to SCFA, kidney-residing olfactory receptor 78 (Olfr78) and G protein-coupled receptor 41 (Gpr41) in the muscular layer of blood vessels individually fine-tune renin expression and give rise to hypertension [74]. Another involved molecule is bile acid, whose metabolism is mediated by a flora-answering farnesoid X receptor (FAR) [87]. These molecules facilitate crosstalk between gut microbiota and host metabolism.

Finally, gut dysbiosis compromises host metabolism. In lipocalin 2 double-knockout mice, MetS was spontaneously provoked and was not further aggravated by the consumption of a high-fat diet [75]. Some human studies have attributed obesity to a combination of reduced bacterial populations of anti-inflammatory Bifidobacterium, butyrate-producing Akkermansia muciniphila, Actinobacteria, and Bacteriodes, in addition to the dominant presence of *Escherichia coli*, Staphylococcus aureus, Enterobacteriaceae, as well as pathogenic Campylobacter and Shigella [79]. Pyrosequencing performed by Le Roy et al. also proposed that upregulated Lachnospiraceae and Banesiella with downregulated Lactobacilli were predictive of the onset of DM and fatty liver [76]. Amar et al. pinpointed core flora Proteobacteria as a tentative indicator for DM [80]. Metagenomic analysis argued that quantitative diversity and the functional alteration of gut microbiota synergistically determine metabolic phenotype [88]. Recognition of the characteristics of gut micriobiota plays an important role in the development of treatment modalities to enhance prognosis [81]. In summary, endotoxemia, inflammatory predicament, small molecule signaling disruptions, and unbalanced composition of gut flora play primary roles in the pathogenesis of MetS.

## 6. Novel Treatments

Enhanced dissection of the molecular configurations has augmented the advancement of therapeutic schemes. In contrast to the conventional one-for-all management protocols, precision medicine designs individualized recipes by taking personal molecular features into account. Traditional non-pharmaceutical approaches against MetS included diet modification, aerobic exercise, and psychological management, whereas medication was administered primarily for the prevention or treatment of comorbidities. We proposed two novel treatment modalities, fecal microbiota transplantation (FMT) and targeting end products of cholesterol catabolism (Table 4).

Given its established role in MetS, gut microbiota is also an eligible therapeutic target. In the rodent model, administration of Lactobacillus fermentum 296 for a month not only successfully restored the abundance of this species in the intestine, but also rescued the molecular expression of MetS [89]. Consumption of probiotics was illustrated to attenuate insulin resistance and DM in mice feeding on high fat diet [90]. In addition to direct supplementation of microspecies, FMT was introduced to potentially reorganize endogenous bacterial formation. Targeting the end products of cholesterol catabolism is another potential means to antagonize MetS. Accordingly, approaches to manipulate the physiology of bile acid may impede MetS. In the following section, these two aspects will be discussed in detail.

FMT attempts to adjust the microbial composition inside the gastrointestinal tract of the recipient by introducing parts of processed feces from a healthy donor [91]. In the past few decades, FMT was used to tackle a spectrum of clinical settings, from pseudomembrane colitis by recurrent Clostridium difficile infection [92] to inflammatory bowel disease. Other anecdotal reports have also suggested a positive effect of extraintestinal therapeutic applications [93]. Despite these successes, employing FMT in the attempt to manage obesity was initially unsuccessful until animal studies confirmed the pivotal role of the microbial signature. This was achieved by MetS. Bäckhed et al., using adult germ-free mice, showed that gut microbiota are a paramount factor in energy harvesting and storage [73]. Inspired by this concept, Di Luccia et al. successfully rescued fructose-induced MetS by introducing a mixture of ampicillin plus neomycin or oral gavage of fecal samples from control counterparts. Inflammation and oxidative stresses were simultaneously reduced by decreasing the abundance of Coprococcus and Ruminococcus [73]. Subsequently, Smits et al. randomly assigned 20 obese and insulin-resistant males with MetS to receive FMT either from single lean vegan donor or autologously [94]. FMT samples harvested from the former successfully modified the composition of gut microbiome. However, the trimethylamine-N-oxide pathway remained unchanged. Oral administration of the exogenous microbiota also failed to abate the expressions of those biomarkers for vascular inflammation. Although allogenic FMT was demonstrated effective in enhancing small intestine permeability in patients with nonalcoholic fatty liver disease [95], no randomized trial has examined FMT efficacy on MetS to date, and relevant human studies are scarce. Vrieze et al. documented improved insulin sensitivity and increased butyrate-producing gut bacteria, i.e., Roseburia intestinalis and Eubacterium hallii, in individuals with MetS after receiving FMT from lean donors as compared with autologous flora infusion as placebo [96]. Zheng et al. systematically reviewed the efficacy of FMT on MetS, demonstrating pronounced improvement in insulin sensitivity and reduced HbA1C levels [97]. On the other hand, other clinical parameters, e.g., BMI, fasting plasma glucose, and triglyceride, showed no significant differences. Long term clinical parameters have not been assessed. To ensure optimized efficacy, the concept of individualized FMT was proposed, as one ‘magic bullet’ was regarded infeasible to manage heterogeneous phenotypes of MetS. Achieving precision medicine also necessitated the consideration and manipulation of baseline clinical characteristics of the recipients. For example, alleviating the initial inflammatory condition improved the engraftment of FMT [98]. Moreover, Alang et al. noticed weight gain in some patients after FMT, and argued that the body composition of the donor matters as well [99]. Thorough consideration of the benefits verses potential adverse effects of FMT in the treatment of MetS were thus mandated. On the other hand, careful screening and selection of appropriate donor remains pivotal to ensure treatment efficacy. Specific microbiota features were recognized in respective clinical scenarios [100]. Extensive collection of the stool samples to establish a FMT bank, in addition to the standardization of executive protocols, forms the basis for this novel MetS treatment modality. Future research on the preparation, dosing, and delivery of fecal material is needed to ensure successful engrafting and anticipated outcomes.

Another aspect of the consequences from gut dysbiosis in MetS involves the regulation of bile acid [101]. Secreted by the liver as the end product of cholesterol catabolism, bile acids participate in intestinal nutrient absorption, and are determinants for gut microbiota growth. Targeting the production and signal transduction pathway of bile acid also has therapeutic implications for MetS. Extensive research has set out to define the functional role of bile acid against MetS, not only as it is closely intertwined with gut microbiota, as it has bacteriostatic effects and mucosal protection in the small intestine [102], but also due to its integrative role in lipid synthesis. The literature has discussed the correlation between bile acid and obesity. Preferentially through 12α-hydroxylation, enhanced synthesis and obtuse postprandial elevation of bile acid was observed in obese subjects [103].

Elevation in serum bile acid was found to be associated with hypertriglyceridemia [104]. Figge et al. further described the gut-liver axis. A total of 25 previously healthy subjects who followed fast food diets manifested elevated serum bile acid levels, markers of hepatic injury, and impacted metabolic panels [105]. Bile acid also interacts with both FXR and Takeda G-protein receptor-5 (TGR5) [106]. In conjunction with bile acid anabolism and enterohepatic circulation, utilization of host energy is impacted by these downstream responders in an overlapping fashion. FXR epigenetically causes weight loss, insulin responsiveness, and reduces triglyceride levels, whereas TGR5 raises insulin sensitivity and maintains energy homeostasis [107]. After the secondary bile acid, i.e., deoxycholic acid (DCA), became known for its effect in promoting fat absorption and cholesterol excretion, the prototypical use of DCA was initially employed for cosmetic purposes to shrink submental adipose tissue [108]. Subsequent studies further proposed several previously unknown contributors as respective or dual activators on FXR and TGR5 against MetS. A detailed review is available elsewhere [109]. In addition, the modulating effect on circadian rhythm by bile acid metabolism has been well-established. Normally, a small heterodimer partner regulates the level of serum bile acid, which drops to lowest at midday while peaks at the beginning and the ending of the dark phase [110]. Disturbed lifestyle and irregular diet habits disrupt circadian rhythm, rattle gluconeogenesis, lipogenesis, and bile acid metabolism, ultimately leading to MetS [111]. However, the side effects of medications which manipulate bile acid metabolism, such as pruritus, remain problematic as they perpetuate noncompliance. Future recognition and minimization of adverse events through tissue-specific interventions may pave the way for clinical use of bile acid derivatives and drug development against MetS.

## 7. Conclusions

As lifestyle changes have taken place in the contemporary era, MetS has become a prevalent issue of concern. The clinical burden of this condition has been particularly pronounced during the SARS-CoV-2 pandemic. The evaluation of host characteristics has become a prerequisite to developing precision medicine and improving prognosis. Thorough investigation of the molecular composition of MetS as a disease entity is underway via metabolomics and proteomics, which enable the identification of individualized therapeutic targets. Delineation of biochemical dynamics facilitates the monitoring of disease progression and increases treatment efficacy. Clear depictions of gut microbiota and inflammatory status may further elucidate the pathogenesis of MetS. Evidence on the use of FMT to antagonize metabolic disorders by rebalancing gut microbiota and manipulating bile acid synthesis is underway, and represents a promising area of research. In the foreseeable future, MetS management will experience a paradigm shift, as research from both top-down and bottom-up investigations continues to uncover the key players in its pathogenesis and potential target for treatment.

## Figures and Tables

**Table 1:** Study of metabolomics. AOSD, adult-onset still disease; BCAA, branched-chain amino acid; MetS, metabolic syndrome; PE, phosphatidylethanolamine; and PC, phophatidylcholine.

Author, Year	Species	Cohort	Findings	Ref
Sampey, 2012	Animal	Male Wistar rats	Fat, sodium, and cholesterol in cafeteria diet contribute to MetS.	[37]
Wang, 2011	Human	2422 nondiabetic individuals in Framingham Offspring Study	Metabolism of amino acid participates in the pathogenesis of diabetes.	[34]
Surowiec, 2019		115 Dutch from Leiden Longevity Study	Valeryl carnitine, pyruvic acid, lactic acid, alanine, and diglyceride are connected to MetS.	[38]
Warmbrunn, 2021		132 treatment-naïve males with MetS	Androgen, fatty acid, PE, and PC pathways are the metabolites related to cardiovascular disease.	[39]
Gall, 2010		399 nondiabetic participants	Alpha-hydroxybutyrate was significantly correlated with glucose metabolism	[40]
Zhong, 2017		69 obese subjects (26 with MetS)	Metabolomics distinguishes patient with MetS from mere obesity.	[41]
Floegel, 2014		2380 Europeans	Homeostasis of hepatic phospholipid was associated with cardiopulmonary fitness	[42]
Libert, 2018		90 adults	Worsening health upregulates BCAAs, aromatic amino acids, lysine, and alpha-aminoadipate.	[43]
Chen, 2016		32 AOSD subjects and 30 healthy controls	13 metabolites differently expressed in AOSD are associated with MetS.	[44]
Capel, 2020		298 subjects from French MONA LISA trial	Serum BCAA, gamma-glutamyl amino acids, arginine metabolites, and proline levels are changed in MetS.	[45]
Siopi, 2019		23 sedentary males (9 with MetS; 14 controls)	Exercise downregulates BCAA, alanine, acetylcarnitine, choline, and betaine, antagonizing MetS.	[46]

**Table 3:** Study of gut microbiota. DM, diabetes mellitus; GLP1, glucagon-like peptide 1; GPR41, G protein-coupled receptor 41; JNK, c-Jun N-Terminal Kinases; Lcn2, lipocalin-2; LPS, lipopolysaccharide; MetS, metabolic syndrome; NASH, non-alcoholic steatohepatitis; and SCFA, short-chain fatty acid.

Author, Year	Species	Cohort	Findings	Ref
O’Donovan, 2020	Animal	Landrace porcine with hypertension	Diet and mineral corticoid were exhibited to alter landscape of microbiota, similar to that in MetS.	[67]
Cani, 2007		Male C57BL6/J mice	Bacterial LPS triggers insulin resistance, obesity, and diabetes.	[69]
Hirosumi, 2002		JNK1^−/−^ and JNK2^−/−^ C57BL/6J mice	Inflammation and fatty acid lead to obesity and insulin resistance via JNK.	[70]
Tolhurst, 2012		FFAR2^−/−^ and FFAR3^−/−^ C57B/6 mice	SCFA brings about diabetes via GLP1.	[71]
Samuel, 2008		Gpr41^−/−^ and Gpr41^+/+^ mice	Gut microbiota regulates host energy through Gpr41.	[72]
Bäckhed, 2007		Germ free Fiaf^−/−^ mice	Diet-induced obesity is secondary to modified fatty acid oxidation by intestinal flora.	[73]
Pluznick, 2013		Olfr78^−/−^mice and Gpr41^−/−^mice	Gut microbiota and cardiovascular system cross-talk via Olfr78 and SCFA.	[74]
Singh, 2020		Lcn2^−/−^ C57BL/6 mice	Gut dysbiosis due to Lcn2 deficiency spontaneously evokes MetS.	[75]
Le roy, 2013	Animal/Human	2 donor male, C57BL/6J mice	Gut microbiota independently contribute to non-alcoholic fatty liver disease.	[76]
Cindoruk, 2008	Human	75 subjects with suspected liver disease	*Helicobacter pylori* infection plays a role in NASH.	[77]
Koren, 2011		30 subjects, half with atherosclerosis	Gut flora is correlated with atherosclerotic plaque.	[78]
Le Chatelier, 2013		123 non-obese and 169 obese Danish subjects	Gut genomic diversity stratifies the risk of obesity-related comorbidity.	[79]
Amar, 2011		3280 subjects without baseline diabetes/obesity	Tissue bacteria is connected with the onset of DM in humans.	[80]
Qin, 2012		345 Chinese	Dysbiosis is associated with DM.	[81]

**Table 4:** Treatment modalities of metabolic syndrome. FMT, fecal microbiota transplantation; FXR, Farnesoid X receptor; TGR5, Takeda G-protein receptor-5.

Approach	Items	
Non-pharmaceutical	diet modification, aerobic exercise, psychological management	
Pharmaceutical	Classic	
	Antihypertensive, antidiabetic, antithrombotic, cholesterol lowering agents	
	Novel	
	FMT	(1) Modify composition of gut microbiome; (2) Reduce inflammation and oxidative stress; (3) Improve insulin sensitivity.
	Bile acid	(1) Interact with FXR and TGR5 (weight loss, insulin response, reduce triglyceride level); (2) Promote fat absorption and cholesterol excretion; (3) Modulate circadian rhythm.

## Data Availability

Not applicable.

## References

[1] Expert Panel on Detection, Evaluation, and Treatment of High Blood Cholesterol in Adults (2001). Executive summary of the third report of the national cholesterol education program (NCEP) expert panel on detection, evaluation, and treatment of high blood cholesterol in adults (Adult Treatment Panel III). JAMA.

[2] Grundy S.M., Cleeman J.I., Daniels S.R., Donato K.A., Eckel R.H., Franklin B.A., Gordon D.J., Krauss R.M., Savage P.J., Smith S.C. (2005). Diagnosis and management of the metabolic syndrome: An American Heart Association/National Heart, Lung, and Blood Institute Scientific Statement. Circulation.

[3] Alberti K.G., Zimmet P., Shaw J., IDF Epidemiology Task Force Consensus Group (2005). The metabolic syndrome—A new worldwide definition. Lancet.

[4] Hardy D.S., Racette S.B., Garvin J.T., Gebrekristos H.T., Mersha T.B. (2021). Ancestry specific associations of a genetic risk score, dietary patterns and metabolic syndrome: A longitudinal ARIC study. BMC Med. Genom..

[5] Zhou J.Y., Song M.Y., Park S. (2019). Carbohydrate and sodium intake and physical activity interact with genetic risk scores of four genetic variants mainly related to lipid metabolism to modulate metabolic syndrome risk in Korean middle-aged adults. Br. J. Nutr..

[6] Hershey M.S., Sotos-Prieto M., Ruiz-Canela M., Christophi C.A., Moffatt S., Martínez-González M.Á., Kales S.N. (2021). The Mediterranean lifestyle (MEDLIFE) index and metabolic syndrome in a non-Mediterranean working population. Clin. Nutr..

[7] Saklayen M.G. (2018). The global epidemic of the metabolic syndrome. Curr. Hypertens Rep..

[8] Bitew Z.W., Alemu A., Tenaw Z., Alebel A., Worku T., Ayele E.G. (2021). Prevalence of metabolic syndrome among children and adolescents in high-income countries: A systematic review and meta-analysis of observational Studies. BioMed Res. Int..

[9] Gami A.S., Witt B.J., Howard D.E., Erwin P.J., Gami L.A., Somers V.K., Montori V.M. (2007). Metabolic syndrome and risk of incident cardiovascular events and death: A systematic review and meta-analysis of longitudinal studies. J. Am. Coll. Cardiol..

[10] Mottillo S., Filion K.B., Genest J., Joseph L., Pilote L., Poirier P., Rinfret S., Schiffrin E.L., Eisenberg M.J. (2010). The metabolic syndrome and cardiovascular risk: A systematic review and meta-analysis. J. Am. Coll. Cardiol..

[11] Zhu S., McClure L.A., Lau H., Romero J.R., White C.L., Babikian V., Nguyen T., Benavente O.R., Kase C.S., Pikula A. (2015). Recurrent vascular events in lacunar stroke patients with metabolic syndrome and/or diabetes. Neurology.

[12] Li X., Li X., Lin H., Fu X., Lin W., Li M., Zeng X., Gao Q. (2017). Metabolic syndrome and stroke: A meta-analysis of prospective cohort studies. J. Clin. Neurosci..

[13] Ren H., Wang J., Gao Y., Yang F., Huang W. (2019). Metabolic syndrome and liver-related events: A systematic review and meta-analysis. BMC Endocr. Disord..

[14] Thomas G., Sehgal A.R., Kashyap S.R., Srinivas T.R., Kirwan J.P., Navaneethan S.D. (2011). Metabolic syndrome and kidney disease: A systematic review and meta-analysis. Clin. J. Am. Soc. Nephrol..

[15] Alizadeh S., Ahmadi M., Ghorbani Nejad B., Djazayeri A., Shab-Bidar S. (2018). Metabolic syndrome and its components are associated with increased chronic kidney disease risk: Evidence from a meta-analysis on 11109003 participants from 66 studies. Int. J. Clin. Pract..

[16] Lee M.K., Han K., Kim M.K., Koh E.S., Kim E.S., Nam G.E., Kwon H.S. (2020). Changes in metabolic syndrome and its components and the risk of type 2 diabetes: A nationwide cohort study. Sci. Rep..

[17] (2002). National Cholesterol Education Program (NCEP) Expert Panel on Detection, Evaluation, and Treatment of High Blood Cholesterol in Adults (Adult Treatment Panel III). Third Report of the NCEP Expert Panel on Detection, Evaluation, and Treatment of High Blood Cholesterol in Adults (Adult Treatment Panel III) final report. Circulation.

[18] Aboonabi A., Meyer R.R., Singh I. (2019). The association between metabolic syndrome components and the development of atherosclerosis. J. Hum. Hypertens..

[19] Hess P.L., Al-Khalidi H.R., Friedman D.J., Mulder H., Kucharska-Newton A., Rosamond W.R., Lopes R.D., Gersh B.J., Mark D.B., Curtis L.H. (2017). The metabolic syndrome and risk of sudden cardiac death: The atherosclerosis risk in communities study. J. Am. Heart Assoc..

[20] Carnethon M.R., Loria C.M., Hill J.O., Sidney S., Savage P.J., Liu K. (2004). Risk factors for the metabolic syndrome. Diabetes Care.

[21] Abbate M., Mascaró C.M., Montemayor S., Casares M., Gómez C., Ugarriza L., Tejada S., Abete I., Zulet M.A., Sureda A. (2021). Non-alcoholic fatty liver disease is associated with kidney glomerular hyperfiltration in adults with metabolic syndrome. J. Clin. Med..

[22] Önnerhag K., Dreja K., Nilsson P.M., Lindgren S. (2019). Increased mortality in non-alcoholic fatty liver disease with chronic kidney disease is explained by metabolic comorbidities. Clin. Res. Hepatol. Gastroenterol..

[23] Li B., Yang J., Zhao F., Zhi L., Wang X., Liu L., Bi Z., Zhao Y. (2020). Prevalence and impact of cardiovascular metabolic diseases on COVID-19 in China. Clin. Res. Cardiol..

[24] León-Pedroza J.I., Rodríguez-Cortés O., Flores-Mejía R., Gaona-Aguas C.V., González-Chávez A. (2021). Impact of meta-bolic syndrome in the clinical outcome of disease by SARS-COV-2 in Mexican population. Arch. Med. Res..

[25] Scalsky R.J., Desai K., Chen Y.J., O’Connell J.R., Perry J.A., Hong C.C. (2020). Baseline Cardiometabolic Profiles and SARSCoV-2 Risk in the UK Biobank. medRxiv.

[26] Landecho M.F., Marin-Oto M., Recalde-Zamacona B., Bilbao I., Frühbeck G. (2021). Obesity as an adipose tissue dysfunction disease and a risk factor for infections—Covid-19 as a case study. Eur. J. Intern. Med..

[27] Kuperberg S.J., Navetta-Modrov B. (2021). The role of obesity in the immunopathogenesis of COVID-19 respiratory disease and critical illness. Am. J. Respir. Cell Mol. Biol..

[28] Andrade Silva M., da Silva A.R.P.A., do Amaral M.A., Fragas M.G., Câmara N.O.S. (2021). Metabolic alterations in SARS-CoV-2 infection and its implication in kidney dysfunction. Front. Physiol..

[29] Hardy E., Fernandez-Patron C. (2021). Targeting MMP-regulation of inflammation to increase metabolic tolerance to COVID-19 pathologies: A hypothesis. Biomolecules.

[30] Xiao N., Nie M., Pang H., Wang B., Hu J., Meng X., Li K., Ran X., Long Q., Deng H. (2021). Integrated cytokine and metabolite analysis reveals immunometabolic reprogramming in COVID-19 patients with therapeutic implications. Nat. Commun..

[31] Lindon J.C., Holmes E., Bollard M.E., Stanley E.G., Nicholson J.K. (2004). Metabonomics technologies and their applications in physiological monitoring, drug safety assessment and disease diagnosis. Biomarkers.

[32] Dunn W.B., Bailey N.J., Johnson H.E. (2005). Measuring the metabolome: Current analytical technologies. Analyst.

[33] Johnson C.H., Ivanisevic J., Siuzdak G. (2016). Metabolomics: Beyond biomarkers and towards mechanisms. Nat. Rev. Mol. Cell Biol..

[34] Wang T.J., Larson M.G., Vasan R.S., Cheng S., Rhee E.P., McCabe E., Lewis G.D., Fox C.S., Jacques P.F., Fernandez C. (2011). Metabolite profiles and the risk of developing diabetes. Nat. Med..

[35] Shah S.H., Kraus W.E., Newgard C.B. (2012). Metabolomic profiling for the identification of novel biomarkers and mechanisms related to common cardiovascular diseases: Form and function. Circulation.

[36] Shintu L., Baudoin R., Navratil V., Prot J.M., Pontoizeau C., Defernez M., Blaise B.J., Domange C., Péry A.R., Toulhoat P. (2012). Metabolomics-on-a-chip and predictive systems toxicology in microfluidic bioartificial organs. Anal. Chem..

[37] Sampey B.P., Freemerman A.J., Zhang J., Kuan P.F., Galanko J.A., O’Connell T.M., Ilkayeva O.R., Muehlbauer M.J., Stevens R.D., Newgard C.B. (2012). Metabolomic profiling reveals mitochondrial-derived lipid biomarkers that drive obesity-associated inflammation. PLoS ONE.

[38] Surowiec I., Noordam R., Bennett K., Beekman M., Slagboom P.E., Lundstedt T., van Heemst D. (2019). Metabolomic and lipidomic assessment of the metabolic syndrome in Dutch middle-aged individuals reveals novel biological signatures separating health and disease. Metabolomics.

[39] Warmbrunn M.V., Koopen A.M., de Clercq N.C., de Groot P.F., Kootte R.S., Ter Horst K.W., Hartstra A.V., Serlie M.J., Ackermans M.T., Soeters M.R. (2021). Metabolite profile of treatment-naive metabolic syndrome subjects in relation to cardiovascular disease risk. Metabolites.

[40] Gall W.E., Beebe K., Lawton K.A., Adam K.P., Mitchell M.W., Nakhle P.J., Ryals J.A., Milburn M.V., Nannipieri M., Camastra S. (2010). Alpha-hydroxybutyrate is an early biomarker of insulin resistance and glucose intolerance in a nondiabetic population. PLoS ONE.

[41] Zhong F., Xu M., Bruno R.S., Ballard K.D., Zhu J. (2017). Targeted high performance liquid chromatography tandem mass spectrometry-based metabolomics differentiates metabolic syndrome from obesity. Exp. Biol. Med..

[42] Floegel A., Wientzek A., Bachlechner U., Jacobs S., Drogan D., Prehn C., Adamski J., Krumsiek J., Schulze M.B., Pischon T. (2014). Linking diet, physical activity, cardiorespiratory fitness and obesity to serum metabolite networks: Findings from a population-based study. Int. J. Obes..

[43] Libert D.M., Nowacki A.S., Natowicz M.R. (2018). Metabolomic analysis of obesity, metabolic syndrome, and type 2 diabetes: Amino acid and acylcarnitine levels change along a spectrum of metabolic wellness. PeerJ.

[44] Chen D.Y., Chen Y.M., Chien H.J., Lin C.C., Hsieh C.W., Chen H.H., Hung W.T., Lai C.C. (2016). Metabolic disturbances in adult-onset Still’s disease evaluated using liquid chromatography/mass spectrometry-based metabolomic analysis. PLoS ONE.

[45] Capel F., Bongard V., Malpuech-Brugère C., Karoly E., Michelotti G.A., Rigaudière J.P., Jouve C., Ferrières J., Marmonier C., Sébédio J.L. (2020). Metabolomics reveals plausible interactive effects between dairy product consumption and metabolic syndrome in humans. Clin. Nutr..

[46] Siopi A., Deda O., Manou V., Kosmidis I., Komninou D., Raikos N., Theodoridis G.A., Mougios V. (2019). Comparison of the serum metabolic fingerprint of different exercise modes in men with and without metabolic syndrome. Metabolites.

[47] Nordstrom A., O’Maille G., Qin C., Siuzdak G. (2006). Nonlinear data alignment for UPLC-MS and HPLC-MS based metabolomics: Quantitative analysis of endogenous and exogenous metabolites in human serum. Anal. Chem..

[48] Theodoridis G., Gika H.G., Wilson I.D. (2008). LC-MS-based methodology for global metabolite profiling in metabonomics/metabolomics. Trends Analyt. Chem..

[49] Wu N., Wang W., Yi M., Cheng S., Wang D. (2018). Study of the metabolomics characteristics of patients with metabolic syndrome based on liquid chromatography quadrupole time-of-flight mass spectrometry. Ann. Endocrinol..

[50] Kasprzak-Drozd K., Oniszczuk T., Stasiak M., Oniszczuk A. (2021). Beneficial effects of phenolic compounds on gut microbiota and metabolic syndrome. Int. J. Mol. Sci..

[51] Monnerie S., Comte B., Ziegler D., Morais J.A., Pujos-Guillot E., Gaudreau P. (2020). Metabolomic and lipidomic signatures of metabolic syndrome and its physiological components in adults: A systematic review. Sci. Rep..

[52] Pujos-Guillot E., Brandolini M., Pétéra M., Grissa D., Joly C., Lyan B., Herquelot É., Czernichow S., Zins M., Goldberg M. (2017). Systems metabolomics for prediction of metabolic syndrome. J. Proteome Res..

[53] Mena-Sánchez G., Becerra-Tomás N., Babio N., Salas-Salvadó J. (2019). Dairy product consumption in the prevention of metabolic syndrome: A systematic review and meta-analysis of prospective cohort studies. Adv. Nutr..

[54] Luo Z., Xu W., Zhang Y., Di L., Shan J. (2020). A review of saponin intervention in metabolic syndrome suggests further study on intestinal microbiota. Pharmacol. Res..

[55] Lin X., Wang Q., Sun S., Xu G., Wu Q., Qi M., Bai F., Yu J. (2020). Astragaloside IV promotes the eNOS/NO/cGMP pathway and improves left ventricular diastolic function in rats with metabolic syndrome. J. Int. Med. Res..

[56] Ponomarenko E.A., Poverennaya E.V., Ilgisonis E.V., Pyatnitskiy M.A., Kopylov A.T., Zgoda V.G., Lisitsa A.V., Archakov A.I. (2016). The size of the human proteome: The width and depth. Int. J. Anal. Chem..

[57] Hsieh C.C., Liao C.C., Liao Y.C., Hwang L.S., Wu L.Y., Hsieh S.C. (2016). Proteomic changes associated with metabolic syndrome in a fructose-fed rat model. J. Food Drug Anal..

[58] Benade J., Sher L., De Klerk S., Deshpande G., Bester D., Marnewick J.L., Sieck G., Laher I., Essop M. (2020). The impact of sugar-sweetened beverage consumption on the liver: A proteomics-based analysis. Antioxidants.

[59] Gueugneau M., Coudy-Gandilhon C., Chambon C., Verney J., Taillandier D., Combaret L., Polge C., Walrand S., Roche F., Barthélémy J.C. (2021). Muscle Proteomic and Transcriptomic Profiling of Healthy Aging and Metabolic Syndrome in Men. Int. J. Mol. Sci..

[60] Markova I., Hüttl M., Oliyarnyk O., Kacerova T., Haluzik M., Kacer P., Seda O., Malinska H. (2019). The effect of dicarbonyl stress on the development of kidney dysfunction in metabolic syndrome: A transcriptomic and proteomic approach. Nutr. Metab..

[61] Conceição G., Matos J., Miranda-Silva D., Gonçalves N., Sousa-Mendes C., Gonçalves A., Ferreira R., Leite-Moreira A.F., Vitorino R., Falcão-Pires I. (2020). Fat quality maters: Distinct proteomic signatures between lean and obese cardiac visceral adipose tissue underlie its differential myocardial impact. Cell Physiol. Biochem..

[62] Pla-Pagà L., Guirro M., Gual-Grau A., Gibert-Ramos A., Foguet-Romero E., Catalán Ú., Mayneris-Perxachs J., Canela N., Valls R.M., Arola L. (2020). Proteomic analysis of heart and kidney tissues in healthy and metabolic syndrome rats after hesperidin supplementation. Mol. Nutr. Food Res..

[63] Alfadda A.A., Masood A., Al-Naami M.Y., Chaurand P., Benabdelkamel H. (2017). A proteomics based approach reveals differential regulation of visceral adipose tissue proteins between metabolically healthy and unhealthy obese patients. Mol. Cells.

[64] Sharma S., Yadav S., Chandiok K., Sharma R.S., Mishra V., Saraswathy K.N. (2019). Protein signatures linking history of miscarriages and metabolic syndrome: A proteomic study among North Indian women. PeerJ.

[65] Xu J., Kitada M., Ogura Y., Koya D. (2021). Relationship between Autophagy and Metabolic Syndrome Characteristics in the Pathogenesis of Atherosclerosis. Front. Cell Dev. Biol..

[66] Dabke K., Hendrick G., Devkota S. (2019). The gut microbiome and metabolic syndrome. J. Clin. Investig..

[67] O’Donovan A.N., Herisson F.M., Fouhy F., Ryan P.M., Whelan D., Johnson C.N., Cluzel G., Ross R.P., Stanton C., Caplice N.M. (2020). Gut microbiome of a porcine model of metabolic syndrome and HFpEF. Am. J. Physiol. Heart Circ. Physiol..

[68] Di Murro B., Moretti M., De Smaele E., Letizia C., Lubrano C., Passarelli P.C., D’Addona A., Pompa G., Papi P. (2021). Microbiological Profiles of Dental Implants in Metabolic Syndrome Patients: A Case-Control Study. Antibiotics.

[69] Cani P.D., Amar J., Iglesias M.A., Poggi M., Knauf C., Bastelica D., Neyrinck A.M., Fava F., Tuohy K.M., Chabo C. (2007). Metabolic endotoxemia initiates obesity and insulin resistance. Diabetes.

[70] Hirosumi J., Tuncman G., Chang L., Görgün C.Z., Uysal K.T., Maeda K., Karin M., Hotamisligil G.S. (2002). A central role for JNK in obesity and insulin resistance. Nature.

[71] Tolhurst G., Heffron H., Lam Y.S., Parker H.E., Habib A.M., Diakogiannaki E., Cameron J., Grosse J., Reimann F., Gribble F.M. (2012). Short-chain fatty acids stimulate glucagon-like peptide-1 secretion via the G-protein-coupled receptor FFAR2. Diabetes.

[72] Samuel B.S., Shaito A., Motoike T., Rey F.E., Backhed F., Manchester J.K., Wang L., Chakravarthy M., Ferrannini E. (2008). Effects of the gut microbiota on host adiposity are modulated by the short-chain fatty-acid binding G protein-coupled receptor, Gpr41. Proc. Natl. Acad. Sci. USA.

[73] Bäckhed F., Manchester J.K., Semenkovich C.F., Gordon J.I. (2007). Mechanisms underlying the resistance to di-et-induced obesity in germ-free mice. Proc. Natl. Acad. Sci. USA.

[74] Pluznick J.L., Protzko R.J., Gevorgyan H., Peterlin Z., Sipos A., Han J., Brunet I., Wan L.X., Rey F., Wang T. (2013). Olfactory receptor responding to gut microbiota-derived signals plays a role in renin secretion and blood pressure regulation. Proc. Natl. Acad. Sci. USA.

[75] Singh V., Galla S., Golonka R.M., Patterson A.D., Chassaing B., Joe B., Vijay-Kumar M. (2020). Lipocalin 2 deficiency-induced gut microbiota dysbiosis evokes metabolic syndrome in aged mice. Physiol. Genom..

[76] Le Roy T., Llopis M., Lepage P., Bruneau A., Rabot S., Bevilacqua C., Martin P., Philippe C., Walker F., Bado A. (2013). Intestinal microbiota determines development of non-alcoholic fatty liver disease in mice. Gut.

[77] Cindoruk M., Cirak M.Y., Unal S., Karakan T., Erkan G., Engin D., Dumlu S., Turet S. (2008). Identification of Helicobacter species by 16S rDNA PCR and sequence analysis in human liver samples from patients with various etiologies of benign liver diseases. Eur. J. Gastroenterol. Hepatol..

[78] Koren O., Spor A., Felin J., Fak F., Stombaugh J., Tremaroli V., Behre C.J., Knight R., Fagerberg B., Ley R.E. (2011). Human oral, gut, and plaque microbiota in patients with atherosclerosis. Proc. Natl. Acad. Sci. USA.

[79] Le Chatelier E., Nielsen T., Qin J., Prifti E., Hildebrand F., Falony G., Almeida M., Arumugam M., Batto J.M., Kennedy S. (2013). Richness of human gut microbiome correlates with metabolic markers. Nature.

[80] Amar J., Serino M., Lange C., Chabo C., Iacovoni J., Mondot S., Lepage P., Klopp C., Mariette J., Bouchez O. (2011). Involvement of tissue bacteria in the onset of diabetes in humans: Evidence for a concept. Diabetologia.

[81] Wutthi-In M., Cheevadhanarak S., Yasom S., Kerdphoo S., Thiennimitr P., Phrommintikul A., Chattipakorn N., Kittichotirat W., Chattipakorn S. (2020). Gut microbiota profiles of treated metabolic syndrome patients and their relationship with metabolic health. Sci. Rep..

[82] McGuckin M.A., Linden S.K., Sutton P., Florin T.H. (2011). Mucin dynamics and enteric pathogens. Nat. Rev. Microbiol..

[83] Piya M.K., Harte A.L., McTernan P.G. (2013). Metabolic endotoxaemia: Is it more than just a gut feeling?. Curr. Opin. Lipidol..

[84] Tontonoz P., Spiegelman B.M. (2008). Fat and beyond: The diverse biology of PPARγ. Annu. Rev. Biochem..

[85] Munford R.S. (2016). Endotoxemia-menace, marker, or mistake?. J. Leukoc. Biol..

[86] Reddy P., Lent-Schochet D., Ramakrishnan N., McLaughlin M., Jialal I. (2019). Metabolic syndrome is an inflammatory disorder: A conspiracy between adipose tissue and phagocytes. Clin. Chim. Acta.

[87] Wahlstrom A., Sayin S.I., Marschall H.U., Backhed F. (2016). Intestinal crosstalk between bile acids and microbiota and its impact on host metabolism. Cell Metab..

[88] Qin J., Li Y., Cai Z., Li S., Zhu J., Zhang F., Liang S., Zhang W., Guan Y., Shen D. (2012). A metagenome-wide association study of gut microbiota in type 2 diabetes. Nature.

[89] Cavalcante R.G.S., de Albuquerque T.M.R., de Luna Freire M.O., Ferreira G.A.H., Carneiro Dos Santos L.A., Magnani M., Cruz J.C., Braga V.A., de Souza E.L., de Brito Alves J.L. (2019). The probiotic Lactobacillus fermentum 296 attenuates cardiometabolic disorders in high fat diet-treated rats. Nutr. Metab. Cardiovasc. Dis..

[90] Balakumar M., Prabhu D., Sathishkumar C., Prabu P., Rokana N., Kumar R., Raghavan S., Soundarajan A., Grover S., Batish V.K. (2018). Improvement in glucose tolerance and insulin sensitivity by probiotic strains of Indian gut origin in high-fat diet-fed C57BL/6J mice. Eur. J. Nutr..

[91] Smits L.P., Bouter K.E., de Vos W.M., Borody T.J., Nieuwdorp M. (2013). Therapeutic potential of fecal microbiota transplantation. Gastroenterology.

[92] Kelly C.R., Khoruts A., Staley C., Sadowsky M.J., Abd M., Alani M., Bakow B., Curran P., McKenney J., Tisch A. (2016). Effect of fecal microbiota transplantation on recurrence in multiply recurrent clostridium difficile infection: A randomized trial. Ann. Intern. Med..

[93] Borody T.J., Paramsothy S., Agrawal G. (2013). Fecal microbiota transplantation: Indications, methods, evidence, and future directions. Curr. Gastroenterol. Rep..

[94] Smits L.P., Kootte R.S., Levin E., Prodan A., Fuentes S., Zoetendal E.G., Wang Z., Levison B.S., Cleophas M.C.P., Kemper E.M. (2018). Effect of vegan fecal microbiota transplantation oncarnitine- and choline-derived trimethylamine-N-oxide production and vascular inflammation in patients with metabolic syndrome. J. Am. Heart Assoc..

[95] Craven L., Rahman A., Nair Parvathy S., Beaton M., Silverman J., Qumosani K., Hramiak I., Hegele R., Joy T., Meddings J. (2020). Allogenic fecal microbiota transplantation in patients with nonalcoholic fatty liver disease improves abnormal small intestinal permeability: A randomized control trial. Am. J. Gastroenterol..

[96] Vrieze A., Van Nood E., Holleman F., Salojärvi J., Kootte R.S., Bartelsman J.F., Dallinga-Thie G.M., Ackermans M.T., Serlie M.J., Oozeer R. (2012). Transfer of intestinal microbiota from lean donors increases insulin sensitivity in individuals with metabolic syndrome. Gastroenterology.

[97] Zhang Z., Mocanu V., Cai C., Dang J., Slater L., Deehan E.C., Walter J., Madsen K.L. (2019). Impact of fecal microbiota transplantation on obesity and metabolic syndrome—A systematic review. Nutrients.

[98] Danne C., Rolhion N., Sokol H. (2021). Recipient factors in faecal microbiota transplantation: One stool does not fit all. Nat. Rev. Gastroenterol. Hepatol..

[99] Alang N., Kelly C.R. (2015). Weight gain after fecal microbiota transplantation. Open Forum Infect Dis.

[100] Bibbò S., Settanni C.R., Porcari S., Bocchino E., Ianiro G., Cammarota G., Gasbarrini A. (2020). Fecal microbiota transplantation: Screening and selection to choose the optimal donor. J. Clin. Med..

[101] Jia W., Wei M., Rajani C., Zheng X. (2021). Targeting the alternative bile acid synthetic pathway for metabolic diseases. Protein Cell.

[102] Li T., Chiang J.Y. (2014). Bile acid signaling in metabolic disease and drug therapy. Pharmacol. Rev..

[103] Haeusler R.A., Camastra S., Nannipieri M., Astiarraga B., Castro-Perez J., Xie D., Wang L., Chakravarthy M., Ferrannini E. (2016). Increased bile acid synthesis and impaired bile acid transport in human obesity. J. Clin. Endocrinol. Metab..

[104] Yang Q., Guo X., Chen Y., Zhang W., Ren J., Wang J., Tang N., Gao A. (2020). Blood levels of perfluoroalkyl substances, elements and their associations with metabolic syndrome in Chinese male adults mediated by metabolic-related risk factors. Sci. Total Environ..

[105] Figge A., Sydor S., Wenning C., Manka P., Assmuth S., Vilchez-Vargas R., Link A., Jähnert A., Brodesser S., Lucas C. (2021). Gender and gut microbiota composition determine hepatic bile acid, metabolic and inflammatory response to a single fast-food meal in healthy adults. Clin. Nutr..

[106] Stepanov V., Stankov K., Mikov M. (2013). The bile acid membrane receptor TGR5: A novel pharmacological target in metabolic, inflammatory and neoplastic disorders. J. Recept. Signal Transduct. Res..

[107] Stanimirov B., Stankov K., Mikov M. (2015). Bile acid signaling through farnesoid X and TGR5 receptors in hepatobiliary and intestinal diseases. Hepatobiliary Pancreat. Dis. Int..

[108] Mullard A. (2015). 2014 FDA drug approvals. Nat. Rev. Drug Discov..

[109] Lazarević S., Đanić M., Goločorbin-Kon S., Al-Salami H., Mikov M. (2019). Semisynthetic bile acids: A new therapeutic option for metabolic syndrome. Pharmacol. Res..

[110] Zhang T., Yu F., Guo L., Chen M., Yuan X., Wu B. (2018). Small heterodimer partner regulates circadian cytochromes p450 and drug-induced hepatotoxicity. Theranostics.

[111] Huang J., Iqbal J., Saha P.K., Yang Q., Guo X., Chen Y., Moore D.D., Wang L. (2007). Molecular characterization of the role of orphan re-ceptor small heterodimer partner in development of fatty liver. Hepatology.

